# Putrescent Pulps and Their Treatment

**Published:** 1904-02

**Authors:** Algy F. Strange

**Affiliations:** Springfield, Ill.


### PUTRESCENT PULPS AND THEIR TREATMENT.

The causes of putrescent pulps I have divided into four classes: (1) Mechanical; (2) Thermal; (3) Infection through caries, and (4) Lack of nutrition.

(1) Under mechanical causes may be mentioned blows, biting threads, too rapid movement of teeth in regulating, etc.

(2) Therman. Usually fillings which too closely approximate the pulp without sufficient intermediate lining, although I have known smokers to destroy pulps in seemingly sound teeth from the heat of cigars and pipe stems.

(3) The most common cause is from infection through decay, subjecting the pulp to exposure, irritation, inflammation, hypertrophy, supperation and death.

(4) In a few instances we find the pulp dead wthout any apparent cause. In these cases the health of the patient is generally below par, the nutrient function impaired, and upon closer examination we may find calcific deposits in the pulp chamber or in the pulp itself, or upon the apex or root, cutting off its vascular and nerve supply. I have also noticed a few instances which I could hardly place in either class, where I have concluded that it must be due to infection entering the pulp tissue through the apical foramen.

Under a filling a putrescent pulp will cause a throbbing pain and the tooth becomes unusually sensitive to pressure. The application of heat will increase the pain (by the expansion of the gases), while cold will reduce it, the action on a vital tooth being just the reverse.

There is invariably a septic condition present at the apices of the roots, and frequently a necrotic condition of the pericementum, with possibly a disintegration of the alveolia process.

The decomposed pulp of the deciduous teeth seem to become more vile than those of the permanent. The breath of the little patient has an odor strong enough to permeate the air of the entire operating room. They require much more time and patience, and are more difficult to cleanse and treat than those of adults. Often it is impossible to apply the rubber dam, and owing to the restfulness of the little one it it very hard to exclude the fluids of the mouth.

In adults there is seldom an excuse for not applying the rubber-dam, and the success that follows our efforts is largely due to this one simple step, for we all know that there are numerous specie of bacteria always present in the oral secretions and upon the moist mucous membranes of the mouth, some of which seem to be normal inhabitants. The conditions of temperature, moisture and presence of organic pabulum are extremely favorable for their development that it seems impossible to destroy or to remove them.

The important step in the treatment of such canals is the careful removal of the contents, opening up tooth with chisels or large burs, using great care to avoid all pressure that might force contents through apex, thus setting up an acute case of pericementitus.

The contents of the canals should be as nearly sterile as the use of antiseptics will make them before removal. This will require a great deal of care and skill.

In using an antiseptic we should not employ anything of sufficient strength to be of itself a source of irritation, weakening the defensive power of the pericemental tissues, nor anything that would be so coagulative as to block the escape of gases and toxins, thus forcing the septic matter through the apex of root. Something should be used that possesses great penetrative power. All such antiseptics should be applied in such a manner as to permit of gradual absorption into the infected ,area solely through capillary attraction with an entire absence of pressure. For this I should recommend the use of formaline following with an alkali such as hydrogen-dioxide or sodium-dioxide. They, in contact with decomposed organic matter will cause a chemical reaction, with quite a noticeable effervescence, leaving the dirbis in a condition that can easily be washed out. After this careful and thorough cleansing, the canals should be thoroughly dehydrated by means of alcohol and a plentiful supply of as warm air as the patient will bear. The mouths of the tubuil are thus opened so that the fibrellae can be more easily acted upon.

We are now ready for our antiseptic dressing. For this I am now using oxapara with gratifying results. Oxapara is a composition of formaldehyde, thymol, burnt alum and creosote, a powder and liquid which should be mixed to a creamy consistency, and inserted upon a few threads of antiseptic cotton or bituminous paper. Upon the return of the patient, if there has been no pain, I remove the dressing, dropping it into a little peroxide of hydrogen and it there is no effervescence the canals may be filled with safety. As a canal filling I have for some time been employing (in teeth of this condition) a combination of oxapara and carbonized cotton, which is a good quaity of pure antiseptic absorbent cotton saturated with boric acid, placed in an iron retort, which is then slowly raised to a white heat, the cotton being gradually reduced to carbon. This should be held over a busen flame and thorougly sterilized before using as there is no danger of burning it. When dry it readily crumbles and is difficult to work, but in combination with oxapara it works easily and very satisfactorily. If for any reason it should become necessary to remove the canal filling, all that is necessary is to moisten it with the liquid that accompanies the oxapara, which will so soften it that in a few minutes it may be removed with ease. I also insert a gutta-percha point in combination with the carbonized cotton and oxpara when the canal is sufficiently large to permit.

There are occasionally found putrescent pulps which are still quite sensitive upon touch of a broach, which require a quite different treatment before followng out the treatment as just described.

We find that arsenic is of very little service inf urther destroying the sensitivity, as the blood vessels are so engorged and the circulation so impaired that it is not properly absorbed. For such cases a mixture of tanic acid and glycerine will in a few days so toughen and tan the pulp that it may be removed entirely and with little or no pain, after which the treatment would be identical with previous description until ready for filling or crowning, as the case may be.

